# Milky Sap of Greater Celandine (*Chelidonium majus* L.) and Anti-Viral Properties

**DOI:** 10.3390/ijerph17051540

**Published:** 2020-02-27

**Authors:** Joanna Nawrot, Małgorzata Wilk-Jędrusik, Sylwia Nawrot, Krzysztof Nawrot, Barbara Wilk, Renata Dawid-Pać, Maria Urbańska, Iwona Micek, Gerard Nowak, Justyna Gornowicz-Porowska

**Affiliations:** 1 Department of Medicinal and Cosmetic Natural Products Poznan University of Medical Sciences, Fredry 10, 61-701 Poznań, Poland; joannac@ump.edu.pl (J.N.); m.lupus@wp.pl (M.W.-J.); sd.nawrot@gmail.com (S.N.); reniadp@ump.edu.pl (R.D.-P.); murbanska@ump.edu.pl (M.U.); micekiwonax@gmail.com (I.M.); gnowak.gerard@gmail.com (G.N.); 2 Institute of Biosystems Engineering, Faculty of Farming and Bioengineering, Poznan University of Life Sciences, WojskaPolskiego 28, 60-624 Poznań, Poland; krzychu.nawrot@gmail.com; 3 Department of Water and Wastewater Technology, Faculty of Civil and Environmental Engineering, Gdansk University of Technology, 11/12 Narutowicza St., 80-233 Gdansk, Poland; barbara.k.wilk@gmail.com

**Keywords:** *Chelidonium majus*, viral warts, alkaloids, anti-viral properties

## Abstract

The milky juice of the greater celandine herb has been used in folk medicine and in homeopathy for treatment of viral warts for years. However, classical medicine fails to use properties of celandine herbs in treatment of diseases induced by papilloma viruses. Nevertheless, dermatological outpatient clinics are regularly visited by patients reporting efficacy of milky sap isolated from celandine herb in treatment of their own viral warts. Authors of this report decided to analyze the respective world literature in order to critically evaluate the potential for treatment of viral dermal warts using the milky sap of celandine. Moreover, the case of a 4-year old boy was presented, the parents of whom applied the milky sap of celandine on viral warts on hands. Thus, *Ch. majus* may be a potential therapeutic modality for skin warts, especially in a young patients, where conventional therapy may be difficult to apply.

## 1. Introduction

Greater celandine (*Chelidonium majus* L. - *Papaveraceae*) is a perennial plant growing in regions of moderate climate, on the continents of Europe, Asia, North America and in a part of Northwest Africa. In Poland it is found across the entire country. Greater celandine prefers humid soils, rich in nitrogen and calcium [1]. Most frequently it appears as a ruderal weed in shaded sites.

The plant manifests a thick, fusiform, strongly branched root and a thin, forked and sparsely haired stalk, reaching up to 50 cm. The leafage of the celandine herb shows an alternate arrangement. The leaves are pinnate with lobed and wavy-edged margins. Flowers of greater celandine are four-petalled, yellow, radial, in loose corymbs. The phenological period of the blooming phase spans May and September. The fruits manifest the shape of long, cylindrical capsules. All parts of the plant contain a milky sap of yellow-orange color. The flowers are self-pollinating although cross-pollination may also take place. For best cultivation purposes, seeds of greater celandine should best be sown in the autumn [2].

**Figure 1:**
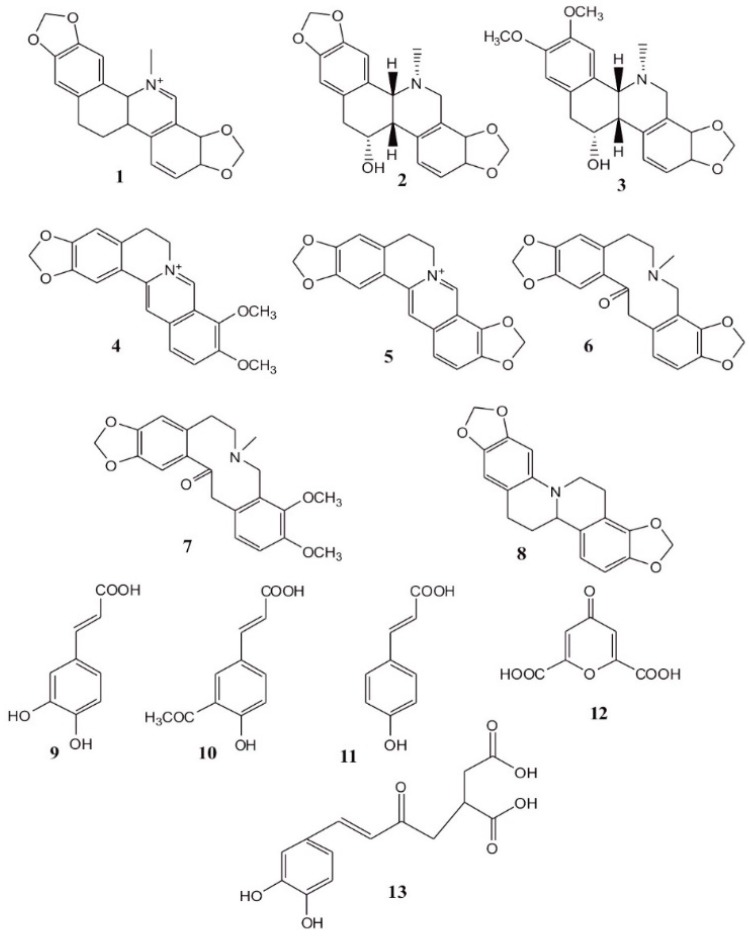
Medically valuable natural compounds of greater celandine: sanguinarine (**1**), chelidonine (**2**), chelerythrine (**3**), protoberberines, berberine (**4**), coptisine (**5**), protopine **(6**), allocryptopine (**7**), stylopine (**8**), caffeic acid (**9**), ferulic acid (**10**), p-coumaric acid (**11**), chelidonic acid (**12**), caffeoyl-malic acid (**13**).

The greater celandine herb is rich in medically valuable natural compounds (Figure 1). Their highest amounts are contained in immature fruits (2.4%) and in roots (4%), while the aerial parts of the plant contain around 0.5% of active compounds. The plant contains, first of all, alkaloids: benzylisoquinolic compounds (0.01%–1%), such as sanguinarine (1), chelidonine (2) and chelerythrine (3) and protoberberines, berberine (4), coptisine (5), protopine (6), allocryptopine (7), stylopine (8). An important group of active compounds involves phenolic acids (caffeic acid (9) and their esters, ferulic acid (10) and p-coumaric acid (11)) as well as chelidonic acid (12) and caffeoyl-malic acid (13) [3,4].

The dried aerial parts of greater celandine collected during flowering are used in medicine. Even if the herb of *Chelidonium majus* is broadly used in herbal therapy, only a few activities were proved by clinical investigations. The principal therapeutic raw material of greater celandine involves the herb [4]. Both raw extracts and purified compounds of *Ch. majus* herb show a broad range of biological activities (anti-inflammatory, anti-microbial, anti-bacterial, anti-viral, anti-mycotic, immunomodulating, anti-neoplastic, cholagogic and analgesic effects), consistent with traditional applications of *Ch. majus* herb [5]. Moreover, the herb was shown to exert cytostatic and cytotoxic effects.

Another raw material of greater celandine, used in traditional herbal therapy, involves the fresh milky sap, the highest concentration of which can be noted in stems and petioles, containing alkaloids, mainly chelidonine [6].

The ability of yellow-orange sap of *Ch. majus* herb to eradicate viral warts may reflect its, proven in many investigations, anti-microbial (including anti-viral) activity, its immunomodulatory effects, and the cytostatic and cytotoxic action of the raw material compounds toward keratinocytes.

Cytotoxic activity is manifested by compounds 1–3, 5, and 6 [7,8,9,10,11]. The growth of keratinocytes is evidently inhibited by sanguinarine, but the effect of chelidonine is much less pronounced. Both chelidonine and sanguinarine in studies in 2007 resulted in apoptosis of MT-4 cells in the course of acute T lymphoblast leukemia [12]. However, they differed in their effect of cell cycle phases: the only chelidonine blocked the cell cycle of MT-4 cells at the threshold of G2/M phases. Even if chelidonine (2), in contrast to sanguinarine (1), does not directly interact with DNA, its effect on the induction of apoptosis slightly exceeds the effects of (1) [12]. Evidence was also provided for induction by chelidonine (2) in the *Chelidonium majus* herb of apoptosis in HeLa cells by activation of signaling pathways involving p38-p53 proteins and AKT/PI3 protein kinase [13]. The effect was also examined of (2) on activity and control of telomerase in HepG2 cells [14]. The alkaloid was found to reduce the activity of telomerase through the reduced expression of hTERT and, therefore, it accelerated the senescence of the cells [12]. The study of Nawrot at al. demonstrated that nucleases CMN1 and CMN2 purified from the milky sap of greater celandine exerted an apoptotic effect on neoplastic cell line HeLa (cells of uterine cervix cancer), but not on CHO cells (ovarian cells of Chinese hamster) [15]. Five alkaloids of *Ch. majus* herbs (1, 2, 6–8) in low concentrations and upon short exposure times demonstrated a pronounced effect on apoptosis of melanoma cells and, in parallel, exerted low toxicity on normal cells [16]. Selective destruction of pathologically altered tissues by milky sap of *Ch. majus* was proved in a clinical study by Isolde Riede [17]. She described two cases of eradication involving preneoplastic lesions and dermal tumors using milky sap of *Ch. majus.* After two days of regular application of the sap on the altered tissues, they altered their color to black. After some time a defect of the tissue developed, covered with a black crust. Before re-applying the milky sap of *Ch. majus*, the crust was removed. After the removal of all the damaged tissues, the site subjected to the treatment healed up.

The immunomodulatory activity of *Ch. majus* was proven in studies of Song et al. [18]. In their *in vitro* investigations, they demonstrated immunomodulatory potential resulting from linking proteins with polysaccharides isolated from *Ch. majus* (CM-Ala) herb, the complex which manifested a mitogenic activity on the spleen, bone marrow cells and which increased levels of granulocyte and macrophage colony-stimulating factor (GM-CSF) [18]. Application of *Ch. majus* extract linked to recombined IFN-gamma was followed by a marked stimulation of NO and TNF-alpha production in mouse peritoneal macrophages [19]. Furthermore, Khmel’nitskaia et al. showed that extract of *Ch. majus* herb improved humoral and cell-mediated immunity and reduced frequency of relapses in pharyngitis affecting children with chronic tonsillitis [20].

Anti-microbial effects of *Ch. majus* herb have been shown in numerous studies. Most broadly, the anti-bacterial effect was described by *Ch. majus* alkaloids. In studies of Leonor and Pinto, anti-bacterial effects of 1, 4 and 5 were demonstrated toward *Bacillus subtilis* [21]. However, compounds 2, 5 and 6 were inactive toward the bacteria [22]. Furthermore, compounds isolated from the over-ground parts of the plant (such as hydroxydihydro and dihydro derivatives of compounds 1 and 3) demonstrated anti-bacterial effects toward methycillin-resistant *S. aureus* [23]. According to Colombo et al., the anti-bacterial effect ascribed to *Ch. majus* herb depends first of all on quaternary ammonium groups of isoquinoline alkaloids. The derivatives and natural compounds free of the quaternary ammonium group manifest an insignificant anti-bacterial activity [7]. Apart from the anti-bacterial effects on Gram-positive bacteria, the antimycotic effects toward *Candida albicans* were proved by Lendfeld et al. [23]. From the point of view involving the treatment of viral dermal warts, the most interesting seems to be the anti-viral effect of *Ch. majus* herb. The anti-viral effect of alkaloids contained in the plant was confirmed in studies of Gerencer et al. of 2006 [24]. They proved the anti-viral activity of *Ch. majus* extracts targeted atretroviruses (HIV-1) [24]. The anti-viral activity of *Ch. majus* herb was also demonstrated by Lozyuk et al., who tested the activity of a Ukraine preparation on Swiss albino mice infected with viruses (herpesvirus, poxvirus, grippe virus) [25]. In some studies, the antiviral activity of *Ch. majus* alkaloids was documented toward human adenoviruses type 5 and 12, herpesvirus and poliovirus [26,27].

Thus, the review of the literature confirms the anti-viral, immunomodulatory, cytostatic and cytotoxic effect of greater celandine herb preparations. There are data reporting [28] the inhibitory effect of *Ch. majus* on HIV virus, adenoviruses, encephalomyocardis virus, and influenza virus type A and B development. The immunomodulatory and cytoprotective effect is probably associated with the alleviation of oxidative stress and the reduction of the level of the proinflammatory cytokines (like TNF-α, IL-6).

Warts are very common, with an incidence of approximately 10% in children and young adults, thus an effective therapy must provide a reduction in pain and improvement in the quality of life. However, a lot of conventional treatments of cutaneous warts are difficult to apply in young patients and may be associated with serious disadvantages (Table 1). In light of this *Ch. Majus* could be a potential therapeutic modality for skin warts without adverse effects.

### Case Presentation

**Figure 2:**
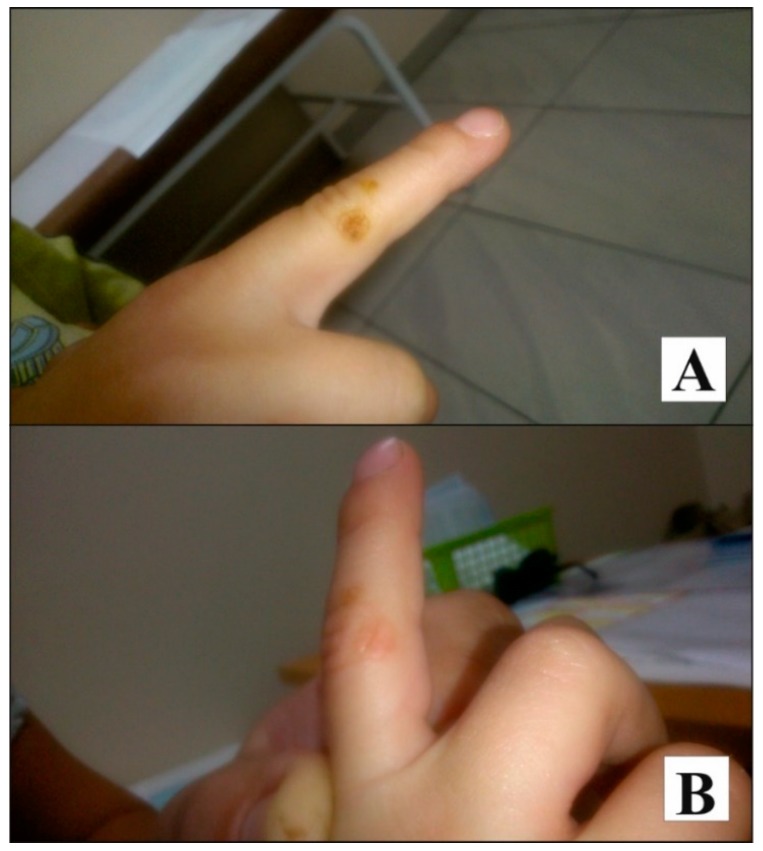
Viral wart on the hand following two weeks of treatment with Chelidonium majus milky sap (**A**). Viral wart on the hand following 3 weeks of treatment with Chelidonium majus milky sap (**B**).

The efficacy of fresh milky sap from *Ch. majus* was also confirmed by the case of a four-year-old boy, who reported with parents to the dermatological outpatient clinic. The fresh juice from the broken stem of the great celandine was rubbed into skin warts (*Ch. majus* were collected from the Botanical Garden at the Department of Medicinal and Cosmetic Natural Products, University of Medical Sciences in Poznan, Poland). The boy, twice a day, applied the fresh yellow-orange sap to the wart on hands which was allowed to dry. Before the next application, the previous dried layer was washed off. After two weeks of treatment, the smaller warts vanished, while the large viral wart on a finger of the hand became flattened by half (Figure 2A). The parents continued the treatment and noted further flattening of the wart (Figure 2B). Following two months, the lesion was cured.

## 2. Discussion

Any attempts, however, of treatment with milky sap of *Ch. majus* require that we are sure that the therapy is safe. Herbal preparations containing alkaloids of *Ch. majus* herb are traditionally used in the treatment of diseases of the gastrointestinal tract and bile ducts. However, numerous scientific investigations prove that the herb of *Ch. majus* manifests a few types of toxicity: cytotoxicity toward tumor cells (which, as described earlier, may be used in the treatment of neoplastic diseases), hepatotoxicity and phototoxicity. The most disquieting seems to be the hepatotoxic action of the plant. Cases of liver damage were described following oral administration of preparations containing *Ch. majus* herb [29,30,31]. However, discontinuation of administration involving celandine herb led to hepatic functions recovering within 2 months and the clinical condition of the patients returned to normal [29]. The caution in internal consumption is necessary for alleged hepatotoxicity (probably due to pharmacological interaction with hormones and non-steroidal anti-inflammatory drugs).

In viral warts, however, the milky sap of greater celandine is applied externally on the skin. No data are available on transdermal absorption of the raw material or its extract. In 2008, the European Council decided that the available data are insufficient to confirm the safety of the extract of *Chelidoniummajus* herb for application in cosmetic products. Moreover, due to the potential for liver damage and very scant information on systemic toxicity it decided to forbid the use of *Ch. majus* extract in all cosmetic products [32]. In parallel, the European Council suggested 28-day NOAEL testing (testing of the highest concentration of a substance at which no undesired effects are noted, while the higher doses or concentrations induce such effects) and suggested a determination of the dermal penetration manifested by celandine herb preparations. In addition, the requirement was indicated to make the extraction methods uniform in order to assure as a low as possible variability in extracts.

Thus, the external application of *Ch. majus* milky sap for the treatment of viral warts remains an open topic, particularly because its transdermal penetration remains to be established. Moreover, a literature of the subject contains studies pointing to the hepatoprotective effects of the celandine herb. The raw material proved to be effective in counteracting hepatic carcinogenesis induced by p-dimethylaminoazobenzene in mice [33]. In addition, studies on rats showed that ethanol extract of *Ch. majus* herb exerts an evident hepatoprotective effect against the damage induced by CCl_4_, reducing the number of necrotic cells and decreasing fibrosis, as well as lowering activities of aminotransferases and bilirubin concentration in blood [34,35].

The phototoxic effect cannot be taken as a contraindication to the application of preparation of a celandine herb. The leaflet should contain information that exposure to the sun should be avoided in the course of treatment with extracts of *Ch. majus* herb.

It should also be analyzed why there exists a group of patients in whom the application of *Ch. majus* milky sap is not followed by the elimination of viral warts. A few hypotheses explaining the problem are possible. First, the treatment of viral warts with celandine milky sap is a prolonged process and it may even take 2 to 3 months. The patients are frequently impatient and following a few applications come to the dermatological outpatient clinic asking to remove the lesion using liquid nitrogen or nitrous oxide. Second, the efficacy of treatment may reflect the time of harvesting the plant. According to studies of Jakovljević et al., concentrations of secondary metabolites in *Ch. majus* herb depend on the phenological phase of plant growth [36]. In the above study, the highest concentration of phenolic compounds was obtained from plants harvested in April, when the plants were at the phase of a rosette. Between May and July, the concentration of phenolic compounds was decreasing (with the lowest value at the phase of blooming) to increase again in August, when the plants enter the phase of fruit formation. The subsequent hypothesis may involve a manifestation in a proportion of patients resistant to treatment, which occurs also in the course of therapies using methods recognized by classical medicine.

The effects of treatment using milky sap of celandine are highly valued by patients, as confirmed by the case of a four-year-old boy, the hand warts of whom vanished following the application of the raw material.

The demand for complementary therapeutics, including herbal medicine, is an emerging trend due to the awareness of potential side effects that synthetic drugs might cause. Thus, studies on the potential for application and safety of the milky sap and extracts of *Ch. majus* in the treatment of viral dermal warts should be continued. Further clinical trials on the comparison of *Ch. majus* and other treatment modalities of warts are warranted. Further studies are required to investigate the mechanism of action of *Ch. majus* and to isolate the active ingredients.

In future studies, the fact should be taken into account that the efficacy of milky sap of celandine may depend on several variables, including production procedures and manner of storage.

## Figures and Tables

**Table 1:** Comparison of various conventional treatments of cutaneous warts and milky sap from *Ch. majus.*

Characteristics	Methods of Cutaneous Warts Therapy				
	Cryotherapy (Freezing)	Peeling (Salicylic Acid)	Laser Treatment	Minor Surgery (Electrosurgery, Excision, Curettage)	Milky Sap from *Ch. majus*
Complication (pain, blister formation, hemorrhage, infection, excessive granulation tissue formation, hyper-/hypo- pigmentation)	+	−	+	+	−
Availability	+	+	−	−	+
Low cost	+	+	−	−	+
Time (a long-term treatment, long recovery periods)	+	+	−	+	−
Lesions may become recalcitrant	−	+	−	−	−

Explanations: “+” – parameter present; “−” – parameter absent.

## References

[1] Biswas S.J. (2013). *Chelidoniummajus* L.-A Review on Pharmacological activities and clinical effects. Glob. J. Res. Med. Plants Indigen. Med..

[2] Weiss R.F. (1980). Lehrbuch der Phytotherapie.

[3] Dewick P.M. (1998). Medicinal Natural Products a Biosynthetic Approach.

[4] ESCOP Monographs (2003). European Scientific Cooperative On Phytotherapy. Chelidoniiherba.

[5] Glica M., Gaman L., Panait E., Stoian I., Atanasiu V. (2010). *Chelidoniummajus*—An integrative review: Traditional knowledge versus modern findings. Forsch. Komplementmed..

[6] Weiss R.F., Fintelmann V. (2000). Herbal Medicine.

[7] Colombo M.L., Bosisio E. (1996). Pharmacological activities of *Chelidoniummajus* L. (*Papaveraceae*). Pharmacol. Res..

[8] Sokoloff B., Saelhof C.C., Takeuchi Y., Powella R. (1964). The antitumor factors present in *Chelidoniummajus* L. Chelidonine an Protopine. Growth.

[9] Kim H.K., Farnsworth N.R., Blomster R.N., Fong H.H. (1969). Biological and phytochemical evaluation of plants. V. Isolation of two cytotoxic alkaloids from *Chelidoniummajus*. J. Pharm. Sci..

[10] Vavrecková C., Gawlik I., Müller K. (1996). Benzophenanthridine alkaloids of *Chelidoniummajus*; II. Potent inhibitory action against the growth of human keratinocytes. Planta Med..

[11] Rogelj B., Popovic T., Ritonja A., Strukelj B., Brzin J. (1998). Chelidocystatin, a novel phytocystatin from *Chelidoniummajus*. Phytochemistry.

[12] Philchenkov A., Kaminsky V., Zavelevich M. (2007). Apoptogenic activity of two benzophenanthridine alkaloids from *Chelidoniummajus* L. does not correlate with their DNA damaging effects. Stoika R. Toxicol. In Vitro.

[13] Paul A., Bishayee K., Ghosh S., Mukherjee A., Sikdar S., Chakraborty D., Boujedaini N., Khuda-Bukhsh A.R. (2012). Chelidonine isolated from ethanolic extract of *Chelidoniummajus* promotes apoptosis in HeLa cells through p38-p53 and PI3K/AKT signalling pathways. Zhong Xi Yi Jie He Xue Bao.

[14] Noureini S.K., Wink M. (2009). Transcriptional down regulation of hTERT and senescence induction in HepG2 cells by chelidonine. World J. Gastroenterol..

[15] Nawrot R., Wotuñ-Cholewa M., Gozdzicka-Józefiak A. (2008). Nucleases isolated from *Chelidoniummajus* L. milky sap can induce apoptosis in human cervical carcinoma HeLa cells but not in Chinese Hamster Ovary CHO cells. Folia Histochem. Cytobiol..

[16] Culp M., Bragina O. (2013). Capillary electrophoretic study of the synergistic biological effects of alkaloids from *Chelidoniummajus* L. in normal and cancer cells. Anal. Bioanal. Chem..

[17] Riede I. (2014). *Chelidonium majus* in der Therapie pramalinger Hautveranderungen. https://www.researchgate.net/publication/260197370_Chelidonium_majus_in_der_Therapie_pramaligner_Hautveranderungen.

[18] Song J.Y., Yang H.O., Pyo S.N., Jung I.S., Yi S.Y., Yun Y.S. (2002). Immunomodulatory activity of protein-Bound polysaccharide extracted from *Chelidoniummajus*. Archives. Pharmacol. Res..

[19] Chung H.S., An H.J., Jeong H.J., Won J.H., Hong S.H., Kim H.M. (2004). Water extract isolated from *Chelidoniummajus* enhances nitric oxide and tumour necrosis factor-Alpha production via nuclear factorkappa B activation in mouse peritoneal macrophages. J. Pharm. Pharmacol..

[20] Khmel’nitskaia N.M., Vorob’ev K.V., Kliachko L.L., Ankhimova E.S., Kosenko V.A., Tymova E.V., Mal’seva G.S., Medvedev E.A. (1998). A comparative study of conservative treatment schemes in chronic tonsillitis in children. Vest. Otorinolaringol..

[21] Pavao M.L., Pinto R.E. (1995). Sensitivity of Bacillus subtilis to water soluble alkaloid extracts from Azores *Chelidonium majus* L. (*Papaveraceae*) roots. Arquipdago. Life Mar. Sci..

[22] Zuo G.Y., Meng F.Y., Hao X.Y., Zhang Y.L., Wang G.C.H., Xu G.L. (2008). Antibacterial alkaloids from *Chelidoniummajus* Linn (*Papaveraceae*) against clinical isolates of methicillin-Resistant *Staphylococcus aureaus*. J. Pharm. Pharmaceut. Sci..

[23] Lendfeld J., Kroutil M., Marsalek E., Slavik J., Preininger V., Simanek V. (1981). Isolation, chemistry and biology of alkaloids from plants of the papaveraceae: Antiinflammatory activity of quaternary benzophenanthridine alkaloids from *Chelidoniummajus*. Planta Med..

[24] Gerencer M., Turecek P.L., Kistner O., Mitterer A., Savidis-Dacho H., Barrett N.P. (2006). In vitro and in vivo anti-retroviral activity of the substance purified from the aqueous extract of *Chelidoniummajus* L.. Antivir. Res..

[25] Lozjuk R.M., Lisnyak O.I., Lozjuk L.V. (1996). Theoretical grounds and experimental confirmation of the antiviral effect of the preparation Ukrain. Drugs Exp. Clin. Res..

[26] Kery R.Y., Horvath J., Nasz I., Verzar-Petri G., Kulcsar G., Dan P. (1987). Antiviral alkaloid in *Chelidoniummajus* L. Acta Pharmaceu. Hungerica.

[27] Horvath J. Antiviral effect of *Chelidonium* extracts. Proceedings of the 13th International Congress of Chemotherapy.

[28] Zielińska S., Jezierska-Domaradzka A., Wójciak-Kosior M., Sowa I., Junka A., Matkowski A.M. (2018). Greater Celandine’s Ups and Downs-21 Centuries of Medicinal Uses of Chelidoniummajus From the Viewpoint of Today’s Pharmacology. Front Pharmacol..

[29] Crijns A.P., de Smet P.A., van den Heuvel M., Schot B.W., Haagsma E.B. (2002). Acute hepatitis after use of a herbal preparation with greater celandine (*Chelidoniummajus*). Ned. Tijdschr. Geneeskd..

[30] Haderman E., van Overbeke L., Ilegems S., Ferrante M. (2008). Acute hepatitis induced by greater celandine (*Chelidoniummajus*). Acta Gastroenterol. Belg..

[31] Moro P.A., Cassetti F., Giugliano G., Falce M.T., Mazzanti G., Menniti-Ippolito F., Raschetti R., Santuccio C. (2009). Hepatitis from greater celandine (*Chelidoniummajus* L.): Review of literature and report of a new case. J. Ethnopharmacol..

[32] Council of Europe (2008). Active Ingredients Used in Cosmetics: Safety Survey.

[33] Biswas J., Bhattacharjee N., Khuda-Bukhsh A.R. (2008). Efficacy of a plant extract (*Chelidoniummajus* L.) in combating induced hepatocarcinogenesis in mice. Food Chem. Toxicol..

[34] Mitra S., Sur R.K., Roy A., Mukherjee A.S. (1996). Effect of *Chelidoniummajus* L. on experimental hepatic tissue injury. Phytother. Res..

[35] Mitra S., Gole M., Samajdar K., Sur R.K., Chakraborty B.N. (1992). Antihepatotoxic activity of *Chelidoniummajus*. Int. J. Pharmacogn..

[36] Jakovljević Z.D., Stanković S.M., Topuzović D.M. (2013). Seasonal variability of *Chelidoniummajus* L. secondary metabolites content and antioxidant activity. EXCLI J..

